# Monthly Summary

**Published:** 1875-04

**Authors:** 


### MONTHLY SUMMARY.

---

*An Unnatural Position of the Head a Cause of Death from Anœsthetics.*—An interesting paper by Dr. G. W. Copeland appeared some time ago (Feb. 26, 1874), in the *Boston Medical and Surgical Journal*, on the "*Styloid Muscles and Anœsthetics.*" In this article the cause of impeded breathing during anæsthesia was attrbuted to the action of the styloid muscles closing the glottis. It was also there shown that the difficulty could always be relieved by simply tilting the head forward so as to relax these muscles, without making traction on the tongue. In a further contribution by the same writer the *Philadelphia Medical Times* of May 30, it is claimed that death from chloroform and other anæsthetics is often caused by an unnatural position of the head, the latter being thrown back and the styloid muscles put upon the stretch. Circumstances in a number of recorded cases of death from anæsthetics are cited which favor this opinion. It is asserted "that all the deaths from nitrous oxi de gas, and a large number of those from other anæsthetics, have taken place while the patients were in a sitting posture, which would allow the head to fall back farther than if they were lying down ; thus favoring the theory that interference with the free action of the lungs may have been the primary cause of death." The cardiac syncope would be of course more readily induced "in patients suffering from shock or fatty degeneration, or already reduced by disease."

Another point advanced is "the importance of elevating the head sufficiently to compel the patient to inhale the anæsthetic through the nares entirely. If deep inspirations be taken through the open mouth, the lungs are inflated instantaneously, and just as rapidly emptied leaving a long interval while no vapor is in the lungs. If the inhalations be through the nerves, it takes a much longer time to inflate the lungs, and a much longer time to empty them, leaving no interval. Now the number of respirations per minute is the same either way ; hence it follows that it will require a longer time to effect anæsthesia through the mouth than through the nose."

---

*Manufacture of Caoutchouc from Milk-weed.*—The *Canadian Pharmaceutical Journal* states that a company, having an authorized capital of $100.000. has recently been formed for the above purpose at London, Ont. In one experiment, one thousand pounds of milk-weed were operated on, and it was found to yield four per cent. of caoutchouc. The process consisted in subjecting the plant to partial decomposition, heating by steam, and then treating by maceration with coal-tar naphtha. The benzine, holding the caoutchouc in solution, was then distilled, when the rubber was finally obtained in a solid form.

---

*A New Operation for Cleft Palate.*—In a recent number of the *The Record* we noticed a modification of an operation for fissure of the bony palate, performed by Sir Wm. Fergusson (and described in *The Lancet*, vol. ii. p. 784), after the failure of the ordinary operation of Langenback. *The Lancet* of June 20, 1874, contains a description of still another improvement by the same surgeon. The operation, as before described, consists in paring the edges of the mucous membrane on each side of the cleft, and then, by means of a chisel, splitting the hard palate on each side, and forcing the two portions of bone thus obtained towards the middle line. The osseous fragments are then bound together in the middle line by two or three silk ligatures, each of which passes through the nasal cavity. The results of all the cases operated upon up to that date were reported at page 298 of the current volume. Although the operation has hitherto been remarkably successful, considering the severity of some of the cases in which it has been employed, it had one drawback. The two bony fragments were liable to become tilted. To obviate this Sir William recently followed this course :—After paring the edges of the mucous membrane, he pierced the hard palate with an ordinary shoemaker's awl in two places on each side of the cleft, close to the margin, in such a manner that the holes on one side of the fissure were directly opposite those on the other side. A seperate silk suture was then passed through each hole on one side, carried into the nasal cavity, and brought into the mouth again through the holes on the opposite side of the cleft. When the sutures where thus secured, the hard palate was divided on each side outside the apertures, by means of the chisel, in the manner described. The silk sutures were then drawn together, and the two fragments of bone brought into gentle apposition. Sir William remarks that since first performing this operation he has found that it had been previously recommended by Dieffenbach —*Med. Record.*

---

*Electric Light.*—A wild machine, intended for the production of light for light houses, gave a light of such intensity, that, when put upon a lofty building, it cast shadows from the flames of the street lamps a quarter of a mile distant upon the neighboring walls. The same light, at two feet from the reflector, darkened senstized photographic paper as much in twenty seconds as the direct rays of the sun at noon on a clear day in March in one minute.—*Popular Science Monthly.*

---

*Chloral Hydrate and Camphor as a Local Application in Neuralgia*—It is said that the intimate mixture of equal parts of chloral hydrate and camphor will produce a clear fluid which is of the greatest value as a local application in neuralgia. Mr. Lenox Browne states, in the *British Medical Journal*, that he has employed it, and induced professional friends to do so, and that in every case it afforded great, and in some instantaneous, relief. "Its success does not appear," he says, "to be at all dependent on the nerve affected, it being equally efficacious in neuralgia of the sciatica as of the trigeminus. I have found it of the greatest service in neuralgia of the larynx, and in relieving spasmodic cough of a nervous or hysterical character." It is only necessary to paint the mixture lightly over the painful part, and to allow it to dry. It never blisters, though it may occasion a tingling sensation of the skin. He has found it also an excellent application for toothache.—[We have used this remedy in several cases of neuralgia of the trigeminus, but with no good result.]—*Med. Record.*

*An Extraordinary Case.*—An extraordinary case was recently brought before the Dublin Pathological Faculty by Professor R. W. Smith, of Dublin University. The disease under which the woman succumbed, whose skeleton he exhibited, was one of rare occurrence, and difficult alike to diagnose, treat, or even name. At the time of her death the woman was forty-five years old. Fifteen years previously she had been sent to jail for some offence, which was probably committed while insane, as shortly afterward she was transferred to a lunatic asylum. During the first ten years of her residence there nothing remarkable about her was noticed, and she was employed in washing the floors, etc. At the end of this period she ceased to be able to work, and remained in bed for the remaining five years of her life, gradually becoming more feeble, and dwindling away in stature until she became about one-half of the height she was originally. She did not complain of any pain; her limbs became coiled up in every possible shape, and she seemed gradually to disappear from off the face of the earth. She died, possibly, from constitutional disease of the osseous system. He (Professor Smith,) however, looked upon the condition of the bones not as a disease, but as a manifestation of an as yet unknown diseased condition. Professor Smith had weighed all the bones individually; the total weight of the skeleton (including the cranium) was two and one half pounds, which equalled about the fourth part of the weight of a child at birth. The bones were extremely light, soft, fragline, and atrophied in every respect. The number of fractures were prodigious. The ribs were in a hundred fragments. The head of the humerus was bent; the fibulæ were curved; the thigh-bones and pelvis were huddled up together; and the bones of the vertebræ thinned and worn away across the front of their bodies. The lower jaw was atrophied and broken into three fragments; the base of the skull was cribriform all through; and he (Professor Smith) believed that if the woman had lived longer not a vestige of a bone in her body would have been left. As to the nature of this disease he (Professor Smith) believed that it was identical with rickets occurring in the adult; and although that opinion might appear heretical to some, yet he was glad to find that in the last volume of Trousseau's Lectures on Clinical Medicine, that distinguished author had expressed his opinion that osteomalacia and rickets were one and the same disease.—*Irish Hospital Gazette.*

---

*Fine Wire.*—Gold and Platinum have been drawn to a "spider line" for the field of a telescope, by coating the metal with silver, drawing it to the finest number, and then removing the silver coating by acid, leaving the almost imperceptible interior wire, which, in a neat experiment in London, was so attracted that a mile's length only weighed a grain.

*Acidity of the Gastric Juice.*—At a meeting of the Biological Society of Paris, MM. Claude Bernard and Lepine both asserted that the results of their recent experiments led them to reaffirm that, contrary to the statements of some German authors, the glands of the stomach do not secrete an acid juice, and that it is only on the surface of the mucous membrane that gastric juice becomes acid.

---

*Lymphatic Tumors of the Face*—Dr. Leoni describes a remarkable sort of affection that occurred in his own person, and the nature of which he can only suspect. One day his right upper lip commenced to swell until it became a tumor of considerable size. He could assign no cause for it, for he had received no injury there, nor had there been previous trouble of any kind. The swelling was not painful, but gave the sensation of great tension, as if it was in a state of erection. At the end of two hours it had disappeared as suddenly as it came. Subsequently, similar swellings occurred on two occasions, once in the same place, and once in the lower lip and chin, lasting in this last case from three to four hours. He tried in vain to reduce them by pressure and manipulation, and finally introduced an exploring needle through the mucous membrane of the mouth, thinking to evacuate the contents, if they were fluid. Nothing, however, escaped. Leoni thought there was no evidence to show that the tumors contained blood. It seemed more likely that they were lymphatic in character, and produced by some obstruction in the course of lymphatic vessels.

We had observed the same affection, in a less degree, in a young woman, at the commencement of her menstrual epoch.—*Nord. Med. Ark.—Nashville Jour. Med. and Surg.*

---

*Actual Cautery.*—When actual cautery is to be used the iron must be heated till it is really of a white heat, and looks almost as white as white paper. If then applied it destroys the part instantaneously, giving no pain ; but it must be removed quickly on the heat decreasing. If and only red hot the agency is intense. The first time I saw the cautery used, on a girl of 14 years, no pain was given ; the second time, on an elderly person [both for fungus of the upper maxillary bone,] the screeching was fearful, till I told the operator his irons were not half hot enough. He requested me to heat them properly, which being done, not a murmur was heard. The last time I saw it, and was opening four or five sinnures in a horse's shoulder. He never flinched, and seemed scarcely aware of what was being done. I would suggest using to obtain the white heat for actual cautery, a large splint blow pipe.—Dr. J. S. Camden.—*Medical Times and Gazette.*

*Headache from Eye-Strain.*—It is well known that headache is a symptom of many intra-occular disorders, but neither in the works on the eye, says Dr. S. Weir Mitchell, nor elsewhere, is it made plain that headache may be for years almost the sole symptom of grave disorders of accommodation or of defects in the orderly action of the external eye muscles. Dr. Mitchell has often seen the sequence, and in cases of chronic headache, insists upon a careful examination of the eye, and has often been rewarded by finding the pain fade away when the optical effect has been duly corrected. The strain caused by the various forms of astigmatism often causes headache; but a light insufficiency of some one of the extra-ocular ball muscles is far more likely to give rise to it. In all of them the headache comes by degrees, and is at first found only following long use of the eyes. By and by, almost any use of the eye causes pain. The over-effort made to correct or accommodated, and converge or diverge the eyes, at first causes pain only on such effort; but at last the teased brain gets to aching when the patient is not trying the eyes, when he is thinking or doing a little mental arithmetic, or the like. Facts like these have often made physicians overlook the eye trouble as the true parent of the pain. When once the intra-ocular or oculo-motor trouble has been relieved, it seems natural to suppose that the headache would at once disappear, but, in fact, it fades away but slowly, and sudden entire relief is rare.—*Med. and Surg. Rep.*

---

*Assaying by the Spectroscope.*—A paper with the above title has been issued by Mr. Outerbridge, detailing a series of experiments made at the U. S. Mint, in Philadelphia, with a view of ascertaining the possibility of determining the value of metals by the spectroscope. The conclusion arrived at, is that

---

assaying by this means is impracticable.

*Tenotomy in the Treatment of Fracture of the Lower Jaw.*—A machinist came under the treatment of Estlander, having sustained a double fracture of the lower jaw from a heavy blow, the lines of fracture on either side passing through the mental foramen. The separated portion of bone was drawn strongly backwards and downwards, and could only be replaced by using considerable force, but it was found impossible to retain it in place, though many different kinds of apparatus were tried. To overcome the difficulty, Estlander divided the tendons of the genio-glossus, genio-hyoid and digastric muscles, after which there was no difficulty in replacing the fragment and keeping it in place. Neither the tongue nor hyoid bone were materially affected, and the patient made a good recovery. —*Nord. Med. Arkiv.*

*Causes of Death from Petroleum Explosion.*—The *Lancet* expresses the opinion that oftentimes, in fatal explosions of petroleum, death is produced instantaneously by shock, combustion and anæsthesia. Petroleum is a mixture of homologues belonging to the marsh gas family, which for many years have been recognized as powerful anæsthetics. Some 15 years ago an attempt was made to introduce one of them, the hydroid of amyl, as a substitute for chloroform.

---

*Hydrogen Alloys.*—Mr. Dumas has communicated to the French Academy some experiments on the hyeratus of mercury or combinations of hydrogen with that metal. These combinations, it is said, so strongly resemble those which constitutes the amalgams of mercury with silver and other white metals, that it is hardly possible to doubt that they are themselves amalgams, and hence that hydrogen is a metal, a fact affrontly indicated by many other amalgams.

---

*Lacto-phosphate of Lime for Carious Teeth.*—Lactic acid and phosphate of lime, mixed into a paste at the time of using, the Cincinnati *Dental Register* commends highly for filling the cavities of teeth. It becomes hard in a short time, and assimilates itself to the tooth, or at least adheres to it closely and protects the nerve-pulp. The Editor of the *Register* is sanguine in regard to its advantages.

---

*Difference in the Composition of Teeth, etc.*—It was stated at the meeting of the American Dental Society of Europe held at Geneva, in Italy, that in Berlin generally, teeth lacked phosphate of lime. The soil lacked it and even hen's eggs have soft shells. In Norway and Sweden the teeth are more dense and less liable to disease. The teeth in England and America are generall better, denser, harder, and have more resisting power to disease than the teeth in Europe generally. In Berlin soft chalky teeth are almost the rule. Loosening and loss of teeth from tartar abound in an extraordinary degree in Lyons. Chloral hydrate used as a dentrifice is very injurious to the gums. In Zurich the teeth of the working people are worse than those of the higher classes. This is ascribed to the food which consists of bread and poor wine. The people of Egypt and Palestine have good teeth and but little toothache. The Romans have goad teeth, which is attributed to the abundance of lime in the water. In the mountains of Berne, where bread, milk and cheese are the staple foods, the teeth are good and free from tartar. In the valleys the reverse holds good. Children in choccolate manufactories have carious teeth.

